# Attention deficit hyperactivity disorder assessment through objective measures: POV glasses and machine learning approach

**DOI:** 10.3389/fpsyt.2026.1785988

**Published:** 2026-03-17

**Authors:** Hakan Kayış, Çınar Gedizlioğlu, Elif Mumcu, Ayşegül Tuğba Hıra Selen, Akın Tahıllıoğlu, Nurhak Doğan

**Affiliations:** 1 Department of Child and Adolescent Psychiatry, Faculty of Medicine, Zonguldak Bülent Ecevit University, Zonguldak, Türkiye; 2 Department of Computer Engineering, İzmir University of Economics, Izmir, Türkiye; 3 Department of Child and Adolescent Psychiatry, Konya City Hospital, Konya, Türkiye; 4 Department of Child and Adolescent Psychiatry, İzmir Bakırçay University, Izmir, Türkiye; 5 Department of Psychiatry and Behavioral Sciences, The University of Texas Health Science Center at Houston Louis A Faillace MD, Houston, TX, United States

**Keywords:** attention-deficit/hyperactivity disorder (ADHD), digital behavioral biomarkers, machine learning, point-of-view (POV) video analysis, pose estimation

## Abstract

**Introduction:**

The diagnosis of Attention-Deficit/Hyperactivity Disorder (ADHD) largely relies on clinical interviews and parent/teacher-report rating scales, which are vulnerable to subjective bias. Therefore, there is an increasing need for objective measures to complement existing assessment approaches. The aim of this study was to objectively quantify children’s body movement during a controlled, semi structured interaction, to examine differences between children with and without ADHD, and to evaluate the cross-sectional discriminative capacity of these movement-based features using machine learning methods.

**Methods:**

This study employed a cross-sectional, observational case–control design including 37 children diagnosed with ADHD and 29 typically developing children aged 7–11 years. Psychiatric diagnoses were established using the DSM-5–based K-SADS PL interview. Video recordings were obtained during a standardized 5-minute instructional interaction using a researcher-worn point-of-view (POV) camera. Body movement measures of the head, upper limbs, and lower limbs were extracted from the video recordings using MediaPipe Pose. Movement data were statistically compared between groups, followed by classification analyses using machine learning algorithms.

**Results:**

The global activity index was significantly higher in the ADHD group compared to the control group (p = 0.003). Regional analyses revealed significant group differences in shoulder, elbow, ankle, foot, and head movements. A significant positive correlation was found between the global activity index and parent-reported hyperactivity scores (r = 0.28, p = 0.025). In the machine learning analyses, the AdaBoost classifier demonstrated the highest performance, achieving an accuracy of 81.82% and a ROC–AUC value of 0.85.

**Discussion:**

This study demonstrates that video-based movement analyses obtained during controlled, semi-structured interactions may capture motor activity patterns associated with ADHD. The findings are expected to contribute to the development of digital behavioral markers that may complement existing clinical assessment approaches in the context of ADHD evaluation.

## 1 Introduction

Attention Deficit/Hyperactivity Disorder (ADHD) is a neuropsychiatric disorder characterized by symptoms of inattention, hyperactivity, and impulsivity (1). Epidemiological data show that the prevalence of ADHD is 7.6% in the 3–12 age group and 5.6% in the 12–18 age group (2). ADHD symptoms are common in school-aged children and may persist into adulthood. A person with inattention may have difficulty staying on task, maintaining focus, and keeping organized. A hyperactive person may move frequently or fidget excessively. An impulsive person may act without thinking or have difficulty with self-control (3).

Longitudinal studies report that hyperactivity and impulsivity are prominent during childhood and adolescence and decrease with age. Attention deficit, however, appears to be more stable and becomes more dominant during adolescence and young adulthood (4). Therefore, a comprehensive developmental and clinical history remains central to the diagnosis of ADHD (5).

ADHD is diagnosed by evaluating the individual’s developmental history, the presence of current symptoms, and the impairment in functioning (1). In children and adolescents, detailed semi-structured interviews are conducted with the patient’s parents and, if possible, the child. DSM-based parent, teacher, and self-report behavior rating scales, general psychopathology scales, and functioning scales are recommended for diagnostic accuracy and treatment planning. Continuity performance tests and neuropsychological tests also provide additional information and are not used alone for diagnostic purposes (6).

Increasing the accuracy of clinical diagnosis is crucial to ensure that individuals who actually have ADHD receive treatment without delay (7). Currently, ADHD assessment relies on clinical interviews and rating scales (8). Although these tools are widely used in clinical practice, they are prone to informant bias and discrepancies between reporters (9, 10). Therefore, there is growing interest in objective diagnostic tools. Studies using video-based methods have shown that children diagnosed with ADHD tend to focus more on irrelevant areas during tasks, have lower task performance, and exhibit increased motor activity (11). Similarly, a webcam-based study demonstrated that activity measures derived from compressed video showed strong correspondence with observed physical movement and provided an objective index of hyperactivity (12). Although these studies have produced promising results, they require children to either wear a device (13) or interact with a device (11, 12).

This study aims to contribute to the literature by examining objective measures obtained during controlled, semi-structured interactions. We focus on behavioral characteristics that emerge during real-time, controlled, semi-structured interactions in children with ADHD. First, we examine whether body movements quantified using MediaPipe differ between children with ADHD and controls. Second, we evaluate whether these movement features provide objective, cross-sectional group-level information that may complement established clinical assessment methods when analyzed using machine learning approaches.

## 2 Methods

### 2.1 Participants and design

This study employed a cross sectional, observational case control design to investigate behavioral differences between children with ADHD and typically developing controls aged 7–11 years. A total of 66 children aged 7–11 years were included in the study, comprising 37 children diagnosed with ADHD (combined presentation subtype) and 29 typically developing control children. The ADHD group consisted of 11 girls and 26 boys, and the control group included 13 girls and 16 boys. All participants were native Turkish speakers and attended mainstream primary education.

Psychiatric diagnoses were established by an experienced child and adolescent psychiatrist using the Schedule for Affective Disorders and Schizophrenia for School-Age Children—Present and Lifetime Version (K-SADS-PL) (14). To obtain complementary information, parents completed a sociodemographic data form. ADHD symptom severity and disruptive behavior profiles were assessed using the DSM-IV–Based Screening and Assessment Scale for Disruptive Behavior Disorders—Parent Form (15), a standardized instrument evaluating ADHD symptoms.

Exclusion criteria for groups included a history of intellectual disability, autism spectrum disorder, major neurological or sensory impairments, current psychotropic medication use, and the presence of severe psychiatric comorbidities other than ADHD. Children in the control group were required to have no current psychiatric diagnosis and no parental reports of clinically significant behavioral or attentional difficulties.

**Figure 1:**
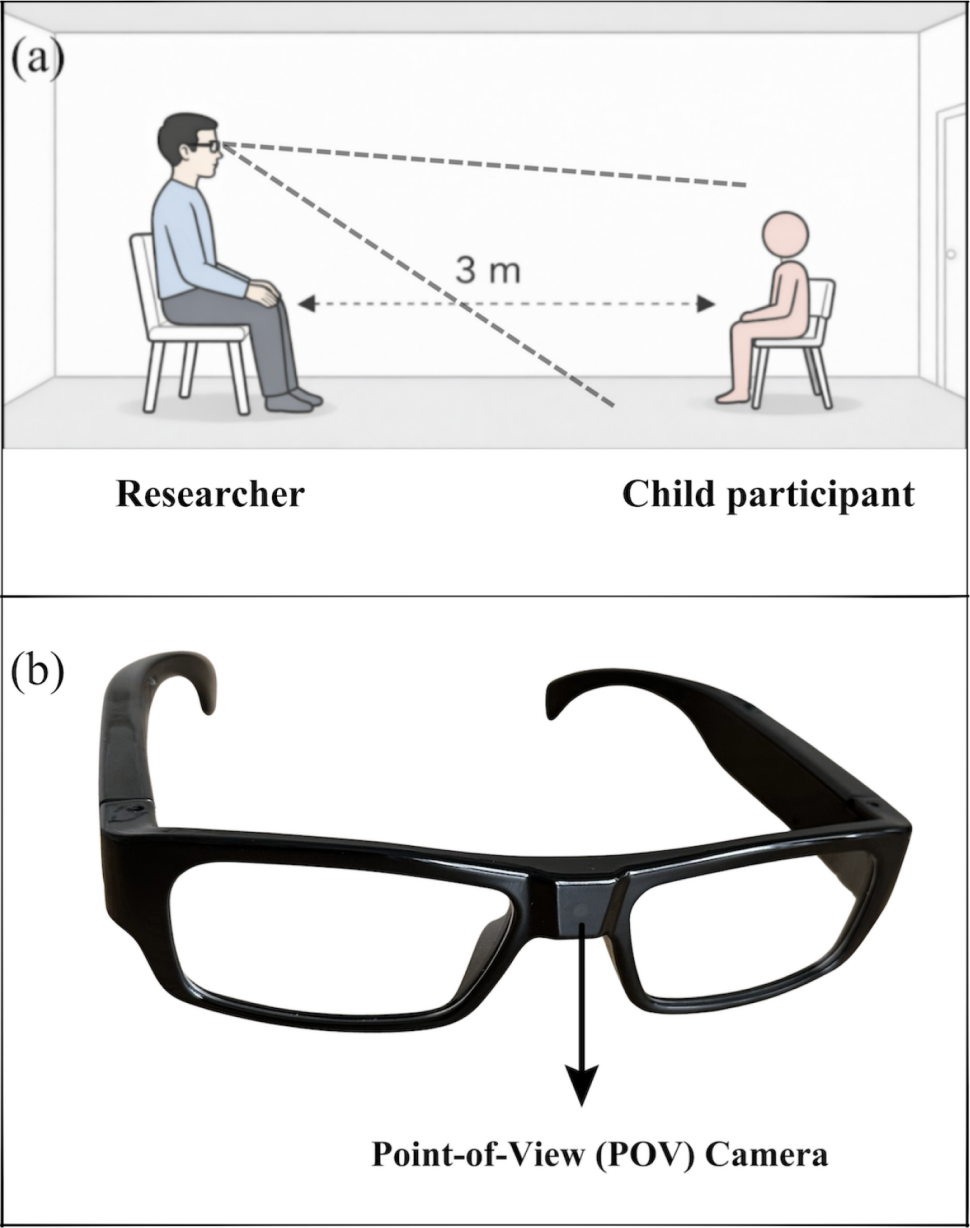
Experimental setup and POV recording device. **(a)** Schematic representation of the standardized semi-structured interaction paradigm used for movement data acquisition. The researcher, wearing POV camera glasses, delivered a standardized 5-minute instructional interaction while seated 3 m from the child participant in a controlled clinical environment. **(b)** POV camera glasses used for first-person video recording during the session.

All sessions were conducted in a hospital-based clinical assessment room under a controlled environment with fixed lighting conditions. A head-mounted point-of-view (POV) camera was worn by the researcher to record the child’s movements. Each child participated in a standardized protocol. The researcher delivered an age-appropriate civic education topic for 5 minutes at a fixed distance of 3 meters (**Figure 1**). To ensure standardization, the researcher wore headphones and followed pre-recorded audio instructions while delivering the content to the child. All videos were recorded at 1920 × 1080 pixel resolution and 30 frames per second, as used in similar clinical interaction settings (16, 17). Video recordings were obtained throughout the session for subsequent analysis. Full-body movement dynamics were extracted from the recordings by a computer engineer blinded to diagnostic grouping. Automated feature extraction was performed using MediaPipe for body and facial landmark detection. The extracted features were then used to differentiate ADHD and non-ADHD groups.

All recordings were made by the same trained researcher (35-year-old male). The researcher completed structured training and pilot sessions on the use of POV glasses before data collection. All sessions were held during daytime hours to minimize fatigue and time related variability.

### 2.2 Instruments

#### 2.2.1 Schedule for affective disorders and schizophrenia for school-age children – present and lifetime version (K-SADS-PL)

In this study, psychiatric diagnoses were assessed using the K-SADS-PL. K-SADS-PL is a semi-structured diagnostic interview widely used in child and adolescent psychiatry to assess psychiatric disorders based on DSM criteria. The interview has been extensively used in both clinical and research settings (14). In the present study, the Turkish DSM-5 version of the K-SADS-PL was used, which has been shown to have good validity and reliability for major childhood psychiatric disorders in Turkish samples (18).

#### 2.2.2 MediaPipe

**Figure 2:**
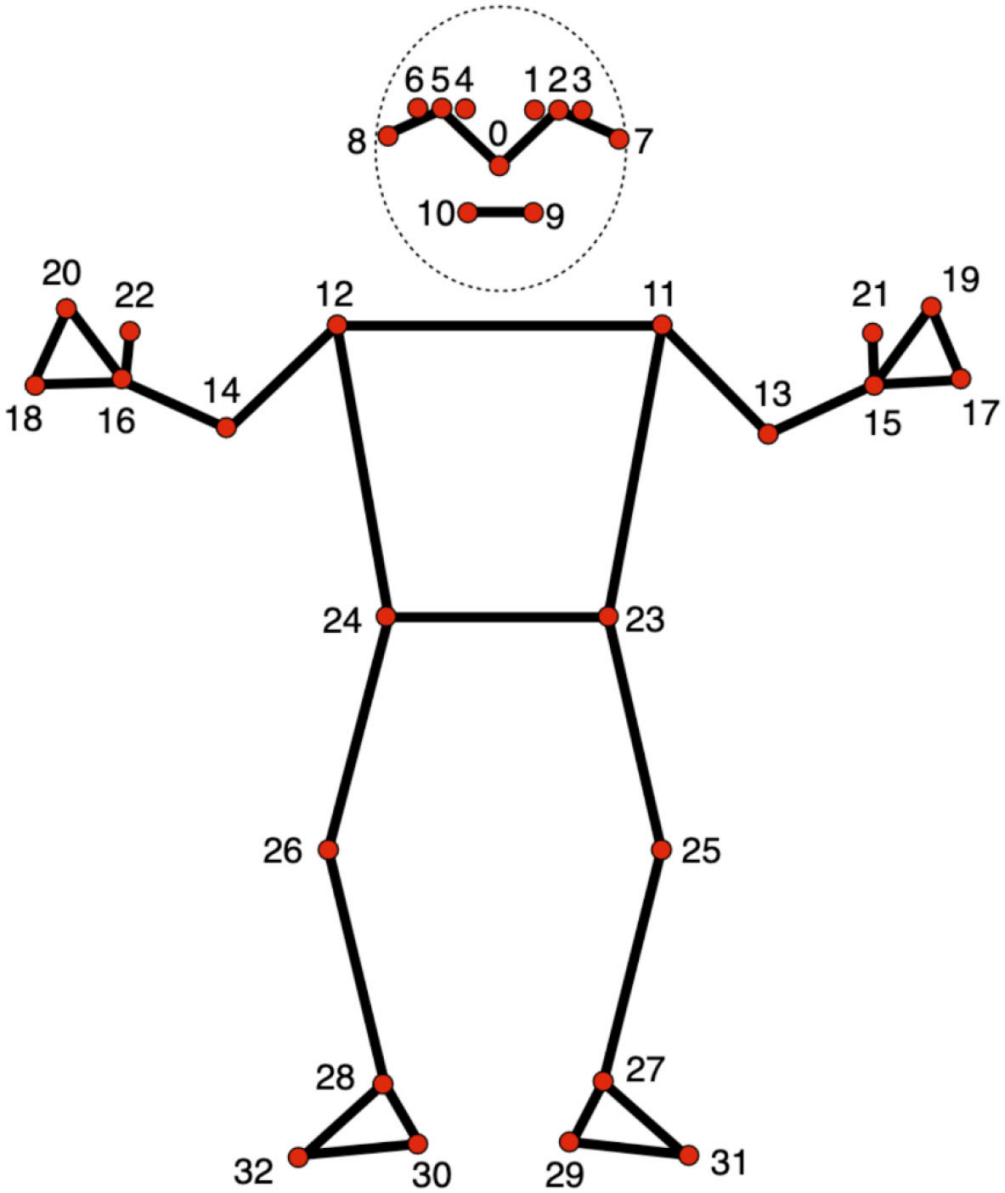
MediaPipe pose landmarks used in the present study (33 keypoints) (19).

**Figure 3:**
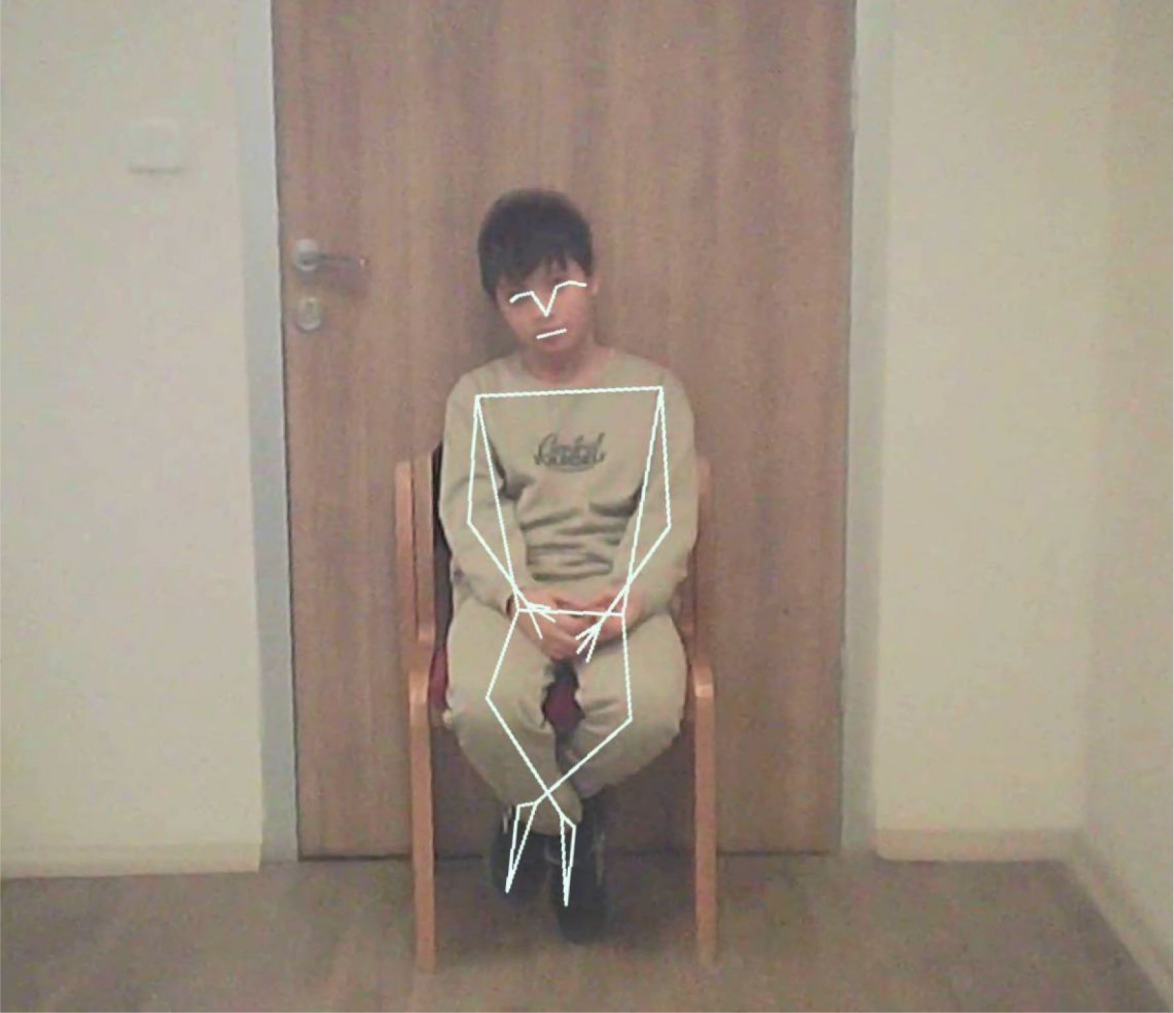
POV camera frame showing MediaPipe Pose landmark detection used for automated movement analysis. Written permission was obtained for the use of the image.

Body movement data were extracted from the video recordings using MediaPipe (version 0.10.11). MediaPipe is a computer-based tool that enables markerless tracking of human body landmarks from videos (19). For each child, the positions of 33 body landmarks were detected across video frames. Changes in these positions over time were used to quantify body movement during the task. In the literature, studies have shown that MediaPipe provides reliable motion tracking performance with acceptable error margins and it is comparable to gold standard motion capture systems (20–22). It has also been used successfully in studies with infants (23) and children across a broad range of ages (24). For each video frame, the MediaPipe Pose Landmarker generated three dimensional (x, y, z) coordinates for 33 anatomical landmarks representing major body joints and segments (**Figure 2**). An example of the pose estimation output overlaid on a recorded frame is shown in **Figure 3**.

#### 2.2.2 DSM-IV–based screening and assessment scale for disruptive behavior disorders

This parent-report scale was developed by Atilla Turgay in 1995 in line with DSM-IV diagnostic criteria. It is used to evaluate symptoms of ADHD as well as other disruptive behavior problems in children and adolescents. The scale includes 41 items covering four symptom domains: inattention (9 items), hyperactivity–impulsivity (9 items), oppositional defiant disorder (8 items), and conduct disorder (15 items). Items are rated on a 4-point scale ranging from 0 to 3, reflecting symptom severity (15). The scale has been validated and shown to be reliable in Turkish samples (25).

### 2.3 Statistical analyses

Statistical analyses were conducted using IBM SPSS Statistics (Version 27). Group differences in demographic variables were assessed using independent-samples t tests for age and chi-square tests for sex. Normality of movement-related variables was examined using the Shapiro–Wilk test. Depending on distributional properties, between-group comparisons were performed using independent-samples t tests or Mann–Whitney U tests. Statistical significance was set at p < 0.05 (two-tailed).

Because movement features are anatomically related, multiple comparison corrections were applied within predefined body region groups. The head domain was represented by a composite head movement index derived from facial landmarks and was analyzed without correction. Upper limb domains included shoulder, elbow, wrist, and hand movements for the right and left sides, with Bonferroni-adjusted significance thresholds (a = 0.0125). Lower limb domains consisted of knee, ankle, and foot movements for the right and left sides, with corrections applied accordingly (a = 0.0167). Hip landmarks were not included in the domains, as movement estimates were calculated relative to a pelvic root. The global activity index was designated as the primary outcome measure and was therefore evaluated without multiple-comparison correction.

Using G*Power 3.1 for a two-tailed independent-samples t-test (α = 0.05, power = 0.80), the minimum required total sample size was 64 participants (32 per group), assuming an effect size of Cohen’s d = 0.72. The final sample included 66 children (37 with ADHD and 29 controls). Although group sizes were unequal, the achieved sample size provided approximately 0.82 power (≈0.816) to detect an effect of this magnitude.

### 2.4 Machine learning analysis

Machine learning methods were applied to evaluate the cross-sectional discriminative capacity of movement features between groups. The feature set consisted of region-specific movement indices derived from pose-based analysis, including the head, bilateral shoulders, elbows, wrists, distal hands, knees, ankles, and distal feet. The global activity index was excluded from machine learning analyses to avoid redundancy as it represents a composite of regional features.

Several supervised learning algorithms were evaluated, including tree-based ensemble methods (Random Forests, Gradient Boosting, Extremely Randomized Trees, and AdaBoost), Support Vector Machines (SVM) and k-Nearest Neighbors (KNN). These models were selected due to their suitability for robustness to nonlinear relationships, and effectiveness in small to moderate sample sizes. Implementations of these models were provided by the Python module scikit-learn (version 1.3.2). Classification performance was assessed using accuracy, precision, recall (sensitivity), specificity, F1-score, and the area under the receiver operating characteristic curve (ROC–AUC).

In the implementation of the Support Vector Machine (SVM) and k-Nearest Neighbors (KNN) classifiers, class imbalance was explicitly addressed by assigning class weights inversely proportional to the number of samples in each class. Minority classes received higher weights and majority classes received lower weights, meaning that misclassification of underrepresented classes resulted in a greater penalty during model training.

All video processing and machine learning analyses were conducted in Python 3.8.6.

### 2.5 Ethics

Ethical approval was obtained from the Non-Interventional Research Ethics Committee of Zonguldak Bülent Ecevit University (decision no. 2025/18). Written informed consent was obtained from the legal guardians of all participating children. Participation was voluntary, and all study procedures were conducted in accordance with the Declaration of Helsinki.

### 2.6 Feature extraction

We aligned video recordings to the onset of the researcher’s first spoken word and trimmed to a fixed duration of 300 seconds (9,000 frames at 30 fps) using Movavi Video Editor (26).

Tracking quality was evaluated based on the confidence scores provided by MediaPipe for each landmark across frames. Frames in which the confidence value fell below 0.50 were marked as invalid for that landmark. Entire recording sessions were excluded if overall missing data exceeded 20% of frames (n=2). For sessions with partial missingness (0–20%), the gaps were reconstructed using linear interpolation for affected landmarks. All landmark positions were smoothed using the One Euro Filter. This is an adaptive low-pass filtering technique designed to reduce high-frequency jitter while preserving rapid, behaviorally meaningful movements (27).

MediaPipe Pose Landmarker produces three-dimensional coordinates; however, depth (z-axis) estimates are less reliable under monocular camera conditions (28). Accordingly, movement quantification in the present study focused on lateral and vertical displacements within the image plane, which provide more stable and reliable estimates. In contrast, z-axis measurements are more susceptible to noise and variability due to camera geometry and occlusion. For this reason, we focused our movement quantification on x–y (lateral and vertical) plane displacements and excluded z-axis (depth) movement from the primary analyses.

For the analyses, we extracted body landmarks aggregated into anatomically meaningful composite points to reduce redundancy. The head is represented by the centroid of 11 facial landmarks (nose, eyes, ears, and mouth), which typically move as a rigid unit. Hand proxies computed as the centroid of three distal hand landmarks (thumb, index, and pinky) for each side, and foot proxies computed as the centroid of the heel and foot-index landmarks for each side. After that, we performed analyses on the resulting set of landmarks: head (11 landmark centroid), left and right shoulder, elbow, wrist, knee, and ankle, as well as left and right hand (3 landmark centroid) and foot (2 landmark centroid). The mapping of the original 33 MediaPipe landmarks to the resulting 15 composite anatomical points is illustrated in **Supplementary Figure 1**.

Movement was quantified using a pelvic root–based coordinate frame, with the root defined as the center of the pelvis, consistent with the literature (29, 30). In this root center usage, all body keypoint coordinates were expressed relative to the pelvic center. This is advantageous in single person and monocular settings, because it reduces the influence of global position shifts and camera relative drift (31). Because all landmark coordinates were expressed relative to the pelvic center, we did not analyze hip landmarks as separate regions in order to avoid circularity and redundancy in displacement computation.

We measured movements by computing Euclidean displacement over short windows. Landmark positions were averaged across consecutive 5 frame windows, and displacement was calculated between these windows. These displacement values then summed across the entire 5-minute recording period to calculate overall motor activity during the protocol. This approach reduces the impact of frame-level jitter inherent to monocular pose estimation and still captures behaviorally meaningful changes in movements. When combined with a pelvic root reference, this further reduces the influence of small camera movements associated with the researcher wearing the POV glasses. The approach was conceptually inspired by the displacement based method used in previous work (32).

## 3 Results

### 3.1 Sample characteristics

The study sample consisted of 66 children, including 37 children with ADHD and 29 typically developing controls. The ADHD and control groups did not differ significantly in age, sex distribution, or height (all p > 0.05; **Table 1**).

**Table 1:** Demographic and clinical characteristics of the study sample.

Variable	ADHD (n = 37)	Control (n = 29)	p value
Age (years), mean ± SD	8.32 ± 1.43	8.79 ± 1.32	0.177
Sex (female/male), n (%)	11/26	13/16	0.206
Height (cm), mean ± SD	130.49 ± 8.54	133.66 ± 7.41	0.118
Inattention score, mean ± SD	15.81 ± 6.93	4.38 ± 4.00	< 0.001
Hyperactivity–Impulsivity score, mean ± SD	16.62 ± 6.07	4.07 ± 3.80	< 0.001

Continuous variables are reported as mean ± SD. Group comparisons for demographic variables were performed using independent-samples t tests. Symptom severity scores were compared using Welch’s t test due to unequal variances. Sex distribution was compared using χ² tests.

As expected, parent-reported symptom severity differed markedly between groups. Children with ADHD showed significantly higher inattention and hyperactivity–impulsivity scores compared to controls (both p < 0.001), confirming the clinical distinction between groups.

### 3.2 Distributional properties of movement measures

Normality of the movement variables was assessed using the Shapiro Wilk test. Many regional movement measures showed non-normal distribution across groups, whereas the global activity index showed a normal distribution; detailed results are presented in **Supplementary Table 1** (**Supplementary Table 1**).

### 3.3 Regional movement differences

Non-parametric group comparisons revealed significant differences in several regional movement measures (**Table 2**). In summary, in the upper limb domains, shoulder and elbow movements differed between groups (left shoulder: U = 301, z = −3.04, p = 0.002; right shoulder: U = 294, z = −3.13, p = 0.002; left elbow: U = 291, z = −3.17, p = 0.002; right elbow: U = 288, z = −3.21, p = 0.001), all remaining significant after Bonferroni correction for the upper limb domains (adjusted a = 0.0125). In contrast, wrist movements did not meet the corrected significance threshold (adjusted a = 0.0125).

**Table 2:** Group differences in regional movement magnitude (Median [Q1, Q3] and Mann–Whitney U tests).

Body region	ADHD median [Q1, Q3] (×10³)	Control median [Q1, Q3] (×10³)	U	Z	Effect size (r)	p (2-tailed)
Head	1.68 [1.33, 2.14]	1.29 [1.16, 1.54]	289.0	−3.20	0.39	0.001
Left shoulder	1.42 [1.11, 1.79]	1.03 [0.96, 1.20]	301.0	−3.04	0.37	0.002 **
Right shoulder	1.44 [1.08, 1.76]	1.05 [0.98, 1.20]	294.0	−3.13	0.39	0.002 **
Left elbow	1.93 [1.46, 2.50]	1.47 [1.28, 1.79]	291.0	−3.17	0.39	0.002 **
Right elbow	1.84 [1.51, 2.41]	1.49 [1.18, 1.68]	288.0	−3.21	0.40	0.001 **
Left wrist	3.17 [2.51, 4.42]	2.71 [1.91, 3.73]	396.0	−1.82	0.22	0.069
Right wrist	3.61 [2.46, 4.45]	2.46 [1.90, 4.25]	382.0	−2.00	0.25	0.046 *
Left hand	3.70 [2.99, 5.22]	3.28 [2.27, 4.76]	412.0	−1.61	0.20	0.108
Right hand	4.12 [2.83, 5.60]	2.89 [2.34, 5.06]	398.0	−1.79	0.22	0.074
Left knee	2.77 [2.39, 3.64]	2.29 [1.77, 2.89]	346.0	−2.46	0.30	0.014 *
Right knee	2.57 [2.17, 3.37]	2.27 [1.69, 2.91]	410.0	−1.63	0.20	0.102
Left ankle	2.63 [1.97, 3.35]	1.76 [1.50, 2.12]	302.0	−3.03	0.37	0.002 **
Right ankle	2.50 [2.12, 2.99]	1.78 [1.53, 2.05]	309.0	−2.94	0.36	0.003 **
Left foot	3.18 [2.31, 4.34]	1.98 [1.58, 2.63]	305.0	−2.99	0.37	0.003 **
Right foot	3.02 [2.38, 3.73]	1.93 [1.67, 2.42]	308.0	−2.95	0.36	0.003 **

Note: * p < 0.05 (uncorrected). ** Significant after domain-specific Bonferroni correction (upper limb a = 0.0125; lower limb a = 0.0167). Head was tested without multiple-comparison correction. Effect size r was calculated as Z/√N (N = 66). Values around 0.1 indicate small, 0.3 medium, and ≥0.5 large effects. Movement values are presented after multiplication by 10³ for readability; statistical analyses were conducted using the original values.

For the lower limb domains, ankle and foot movements showed significant group differences (left ankle: U = 302, z = −3.03, p = 0.002; right ankle: U = 309, z = −2.94, p = 0.003; left foot: U = 305, z = −2.99, p = 0.003; right foot: U = 308, z = −2.95, p = 0.003), which remained significant after Bonferroni correction for the lower limb domains (adjusted a = 0.0167). Knee movements did not reach significance under the corrected threshold.

Head movement also differed between groups (U = 289, z = −3.20, p = 0.001); as the head domain comprised a single outcome measure, no multiple-comparison correction was applied.

The global activity index was significantly higher in the ADHD group (M = 0.0426, SD = 0.0131) compared to the control group (M = 0.0335, SD = 0.0103), t(64) = 3.05, p = 0.003, with a mean difference of 0.0091 (95% CI [0.0031, 0.0150]) and a medium-to-large effect size (Cohen’s d = 0.76). Height-normalized global activity index values remained significantly higher in the ADHD group than in controls t(64) = 3.22, p = 0.002).

As a sensitivity analysis, we repeated group comparisons using height-normalized movement indices; results were consistent with the primary analyses (**Supplementary Table 2**). Normalized values were computed as displacement/height (cm).

#### 3.3.1 Sensitivity analysis: effect of window size

To examine the potential influence of temporal smoothing on the displacement measures, we repeated the regional movement analyses using larger averaging windows (10 and 15 frames). Overall, we observed a pattern of group differences that was similar to the primary 5-frame findings. Across window sizes, bilateral shoulders and elbows, as well as ankle and foot regions, continued to show statistically significant group differences after correction for multiple comparisons. We also found that head movement remained significantly different between groups. Although statistical significance attenuated in some distal upper-limb regions (e.g., wrists and hands) as the window size increased, we observed that the main regional pattern was preserved. Detailed results for the 10- and 15-frame analyses are presented in **Supplementary Tables 3**, **4**, respectively.

### 3.4 Movement variability (standard deviation) analyses

As an additional descriptive analysis, we quantified movement variability by calculating the standard deviation of displacement for each body region. We computed movement variability (SD) from the same 5-frame windowed displacement time series used to derive the activity indices. Children with ADHD showed significantly higher displacement variability compared to typically developing controls across all examined regions (all p values < 0.05). Detailed results are presented in **Table 3**. Given the exploratory and descriptive nature of these variability analyses, no formal correction for multiple comparisons was applied. Movement variability measures were not included in the machine learning feature set and were analyzed separately as descriptive group-level comparisons.

**Table 3:** Group differences in movement variability (standard deviation of displacement).

Body region	ADHD median [Q1, Q3] (×10³)	Control median [Q1, Q3] (×10³)	Mann–Whitney U	p value
Head	1.55 [1.01, 2.08]	1.05 [0.81, 1.58]	356.000	0.020
Left shoulder	1.18 [0.79, 1.68]	0.86 [0.67, 1.06]	317.000	0.005
Right shoulder	1.26 [0.79, 1.77]	0.86 [0.67, 1.08]	306.000	0.003
Left elbow	2.54 [1.35, 3.19]	1.33 [1.08, 1.82]	286.000	0.001
Right elbow	1.92 [1.24, 3.44]	1.40 [0.86, 1.74]	305.000	0.003
Left wrist	4.95 [2.93, 6.07]	3.13 [2.20, 4.28]	323.000	0.006
Right wrist	5.01 [3.39, 6.19]	3.27 [2.81, 4.65]	334.000	0.009
Left hand	5.95 [3.18, 7.15]	3.70 [2.61, 5.31]	346.000	0.014
Right hand	5.86 [4.31, 7.63]	4.06 [3.48, 5.68]	365.000	0.027
Left knee	2.30 [2.01, 3.33]	1.87 [1.31, 2.38]	309.000	0.003
Right knee	2.31 [1.68, 2.99]	1.71 [1.28, 2.36]	368.000	0.029
Left ankle	2.94 [1.71, 3.74]	1.76 [1.17, 2.28]	329.000	0.007
Right ankle	2.94 [1.74, 3.74]	1.93 [1.07, 2.56]	325.000	0.006
Left foot	3.44 [1.72, 4.33]	1.94 [1.13, 2.44]	296.000	0.002
Right foot	3.62 [2.11, 4.17]	2.01 [1.19, 2.66]	304.000	0.003

Values are reported as Median [Q1, Q3]. Group comparisons were conducted using Mann–Whitney U tests due to non-normal distributions. Movement values are presented after multiplication by 10³ for readability; statistical analyses were conducted using the original values.

### 3.5 Association between clinical hyperactivity ratings and objective movement

**Figure 4:**
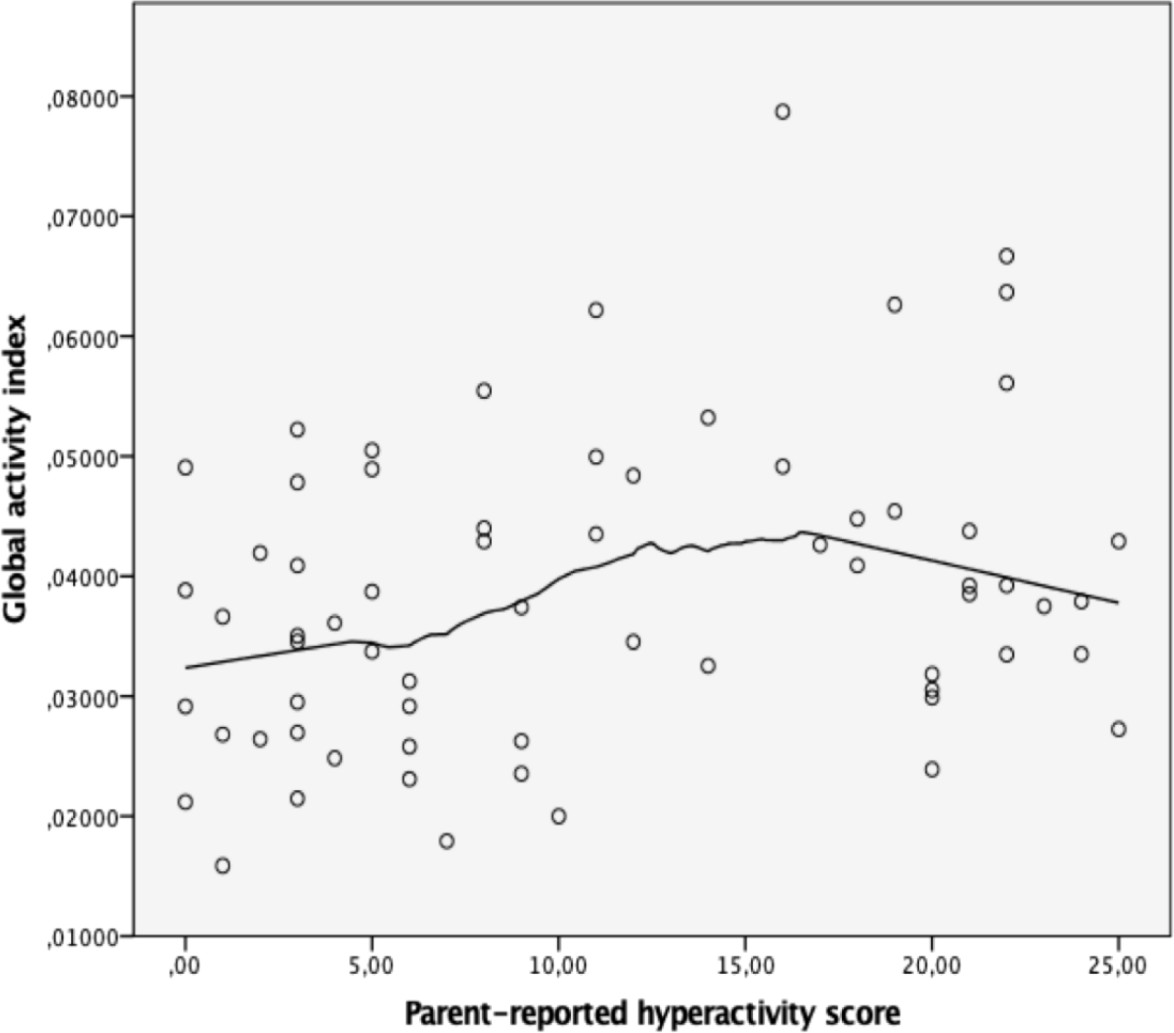
Relationship between parent-reported hyperactivity scores and the global activity index derived from MediaPipe-based movement analysis.

The association between parent-reported hyperactivity scores and objectively quantified movement was examined using Spearman rank-order correlation. Hyperactivity scores showed a positive correlation with the global activity index measured by MediaPipe (ρ = 0.28, p = 0.025, N = 66). When we normalized the movement measures for height, we observed a similar, though slightly weaker, association (ρ = 0.25, p = 0.042). **Figure 4** illustrates the relationship between parent-reported hyperactivity scores and the global activity index.

### 3.6 Anthropometric effects and confounding analyses

Height and age were examined as potential confounding factors. There was no significant group difference in height and age between children with ADHD and controls (**Table 1**). Height was moderately correlated with age (r = 0.68, p < 0.001), and age showed an inverse association with global activity (ρ = −0.34, p = 0.006).

Across the full sample, height showed a modest inverse association with the global activity index (Spearman’s ρ = −0.29, p = 0.017), indicating that greater body height was not associated with increased movement magnitude. To examine whether anthropometric variability confounded group differences, we fitted a linear regression model including group and height as predictors. The overall model was significant (F(2,63) = 6.97, p = 0.002, adjusted R² = 0.155). Group membership remained a significant predictor of global activity after controlling for height (β = 0.31, p = 0.010), while height showed a modest inverse association with movement magnitude (β = −0.24, p = 0.046; **Supplementary Table 5**).

### 3.7 Machine learning analysis

Following statistical analyses, we applied machine learning models to further evaluate the discriminative ability of movement-based behavioral features. We tested multiple supervised classifiers, including tree-based ensemble methods, k-nearest neighbors (KNN), and support vector machines (SVM). Among the evaluated models, the AdaBoost, with a decision tree as its base estimator, demonstrated the highest and most consistent performance across evaluation metrics and was therefore selected as the primary model for reporting classification results.

**Figure 5:**
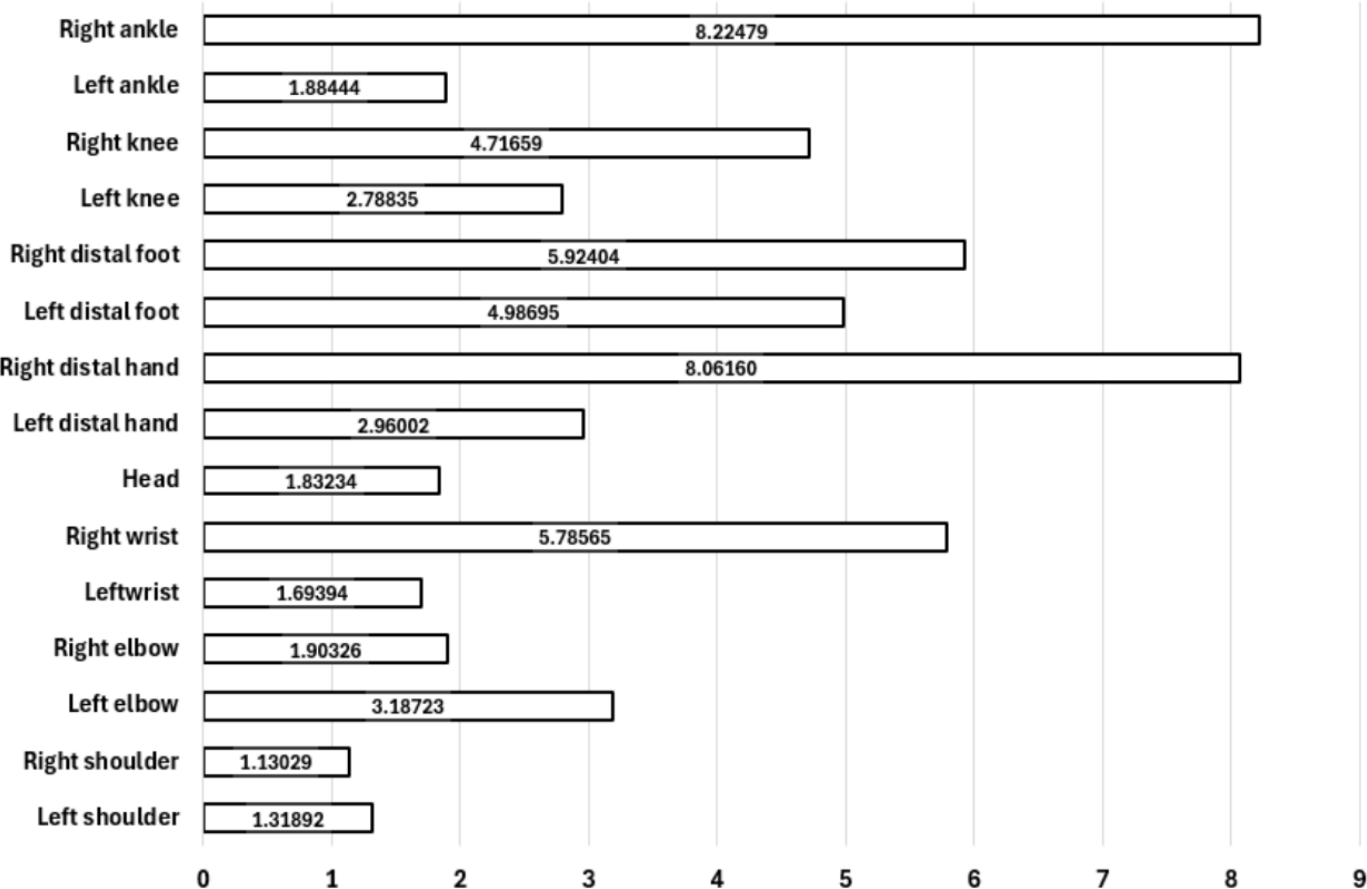
Feature importance scores for movement-based features.

We evaluated feature importance using permutation feature importance (PFI). These computations were performed within the outer loop of a nested cross-validation procedure. Both inner and outer loops of this cross-validation scheme employed a random state of 42 for dataset shuffling. Following completion of the inner cross-validation loop, the model with the optimal set of hyperparameters was selected for feature importance calculations. We performed feature selection within the outer cross-validation loop to prevent information leakage. Here, the values of a single feature are randomly permuted across samples while all other features are kept unchanged. The trained model is then re-evaluated on the validation set. The magnitude of decrease in model performance indicates the importance of the feature. The permutation procedure was repeated multiple times, and importance scores were averaged across repetitions, per feature. Each tested model yielded slightly different importance scores for each feature, which were finally aggregated and reported in **Figure 5**.

After feature importances were calculated, the least important features were iteratively removed from the model. This process continued until the remaining feature set balanced model simplicity and performance, helping to reduce redundancy and mitigate overfitting. The final set of features were the movement-based features for left elbow, right wrist, left distal hand, right distal hand, left distal foot, right distal foot, right knee and right ankle.

**Figure 6:**
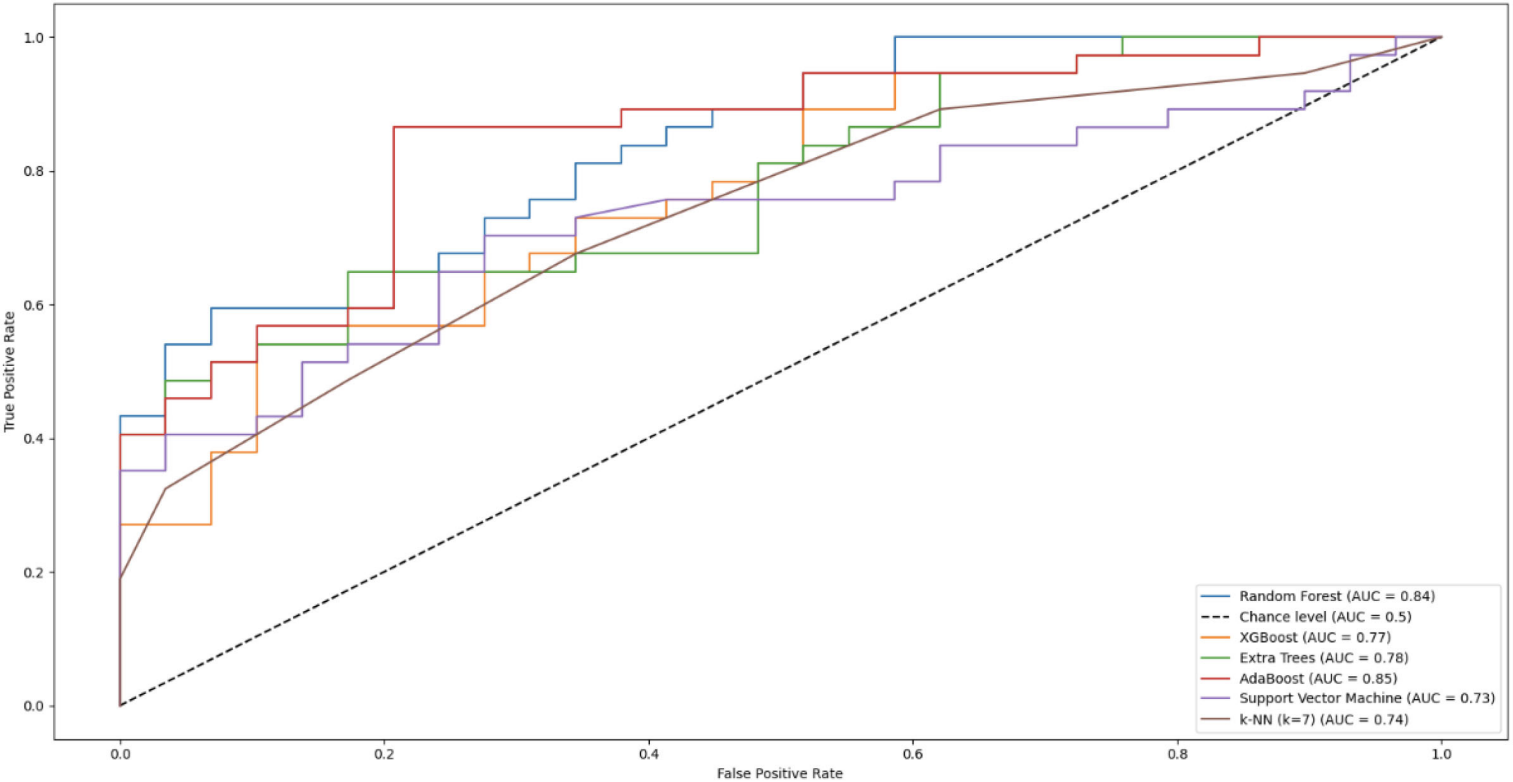
Receiver operating characteristic (ROC) curves illustrating the classification performance of the tested machine learning models. Curves are shown for Random Forest, XGBoost, Extra Trees, AdaBoost, SVM and KNN classifiers. The diagonal dashed line represents chance-level performance (AUC = 0.50).

To examine the cross-sectional discriminative capacity at the group level of the selected features, machine learning models were evaluated using a separate 5-fold cross-validation procedure, with a random state of 42 for shuffling. Performance metrics are reported with Wilson 95% confidence intervals. Among the tested classifiers, the tree-based AdaBoost method achieved the highest performance. Of the 37 positive cases, 31 were correctly classified, and 23 of the 29 negative cases were correctly labeled. The performance metrics of tested classifiers are presented in **Table 4**. Confusion-matrix-based metrics are accompanied by Wilson 95% confidence intervals, while the area under curve (AUC) metric is reported along with the corresponding 95% confidence intervals using the Hanley and McNeil (1982) normal approximation based on pooled outer-fold predictions (N_1_ = 37 ADHD; N_2_ = 29 non-ADHD). See **Figure 6** for ROC curves for all tested classifiers.

**Table 4:** Classification performance metrics. Confusion-matrix-based metrics are reported with Wilson 95% confidence intervals.

Model	Accuracy	Precision	Sensitivity	Specificity	F1 score	AUC
Random Forest	77.27% (65.8-85.7)	78.94% (67.7-87.0)	81.08% (70.0-88.7)	72.41% (60.6–81.7)	80% (68.8-87.9)	0.84 (0.75-0.93)
Extra Trees	72.73% (61.0–82.0)	77.14% (65.7–85.6)	72.97% (61.2–82.2)	72.41% (60.6–81.7)	75% (63.4-83.9)	0.78 (0.67–0.89)
XGBoost	68.18% (56.2-78.2)	68.18% (56.2-78.2)	81.08% (70.0-88.7)	51.72% (39.9-63.3)	74.07% (62.4-83.1)	0.77 (0.66–0.88)
**AdaBoost**	**81.82%** **(70.9–89.3)**	**83.78%** **(73.1–90.8)**	**83.78%** **(73.1–90.8)**	**79.31%** **(68.1–87.3)**	**83.78%** **(73.1–90.8)**	**0.85** **(0.76–0.94)**
SVM	69.70% (57.8-79.4)	75.76% (64.2–84.5)	67.57% (55.6-77.6)	72.41% (60.6–81.7)	71.43% (59.6-80.9)	0.73 (0.61–0.85)
KNN	60.61% (48.5-71.5)	64.10% (52.0–74.6)	67.57% (55.6-77.6)	51.72% (39.9-63.3)	65.79% (53.8–76.1)	0.74 (0.62–0.86)

Bold values represent the performance metrics of the best-performing model (AdaBoost) among the evaluated classifiers.

## 4 Discussion

### 4.1 Principal findings

In this study, we observed that movement features derived from POV video recordings during a brief, semi-structured interaction differed at the group level between children with ADHD and typically developing peers. Using clinician-worn POV recordings combined with automated pose estimation, we identified significant group differences in overall motor activity as well as in head, upper limb, and lower limb movements, despite comparable age and sex distributions. Several regional movement measures, including distal limb segments, remained significant after correction for multiple comparisons, indicating robust and consistent effects. These findings suggest that POV-based, video-driven movement analysis may provide an objective means of quantifying motor activity during controlled, semi-structured interactions, with potential to complement established clinical assessment approaches for ADHD.

In addition to the observed group-level differences, the AdaBoost method achieved the strongest group-level classification performance within this cross-sectional design, with an accuracy of 81.82%, an F1 score of 83.78%, and an area under the ROC curve of 0.85. These results indicate that region-specific movement patterns extracted from brief, semi-structured POV recordings may contain discriminative information at the group level within this cross-sectional sample.

### 4.2 Comparison with prior work

In our study, children with ADHD exhibited significantly higher motor activity during semi-structured interaction compared with controls. Statistically significant increased movement differences were observed in shoulders, elbows, ankles, feet and head movements. When examining the feature importance analysis (**Figure 5**), right-sided body features contributed more strongly to classification performance than left-sided features. Although hand dominance was not assessed in this study, this asymmetry may reflect the higher prevalence of right hand dominance in the population. In the literature, Straczkiewicz et al. reported that activity estimates obtained from accelerometers vary depending on which wrist the device is worn on (33). On the other hand many previous movement-based studies in the ADHD literature have focused on global activity measures (12, 34). Therefore, the observed lateralization should be interpreted with caution.

Various tools have been used for the objective measurement of symptoms related to ADHD. Devices such as wearable sensors and accelerometers have long been used to objectively quantify activity levels (35, 36). Wehrmann and Müller used a webcam-based video activity score and found strong correlations with independent movement ratings (12). A video-based task study reported increased head movements in children with ADHD (11).

Advances in technology have added new tools for the objective assessment of ADHD. For example, in a study conducted by Chang and colleagues, a chair equipped with electrical sensors was used during clinical interviews in a hospital setting. In this study, data obtained from this instrument differed significantly between 31 ADHD patients and 31 control group participants. Machine learning analysis using a support vector machine (SVM) achieved an area under the curve (AUC) of 98% (37). In a similar way to our study Ouyang et al. recorded 4–6 minute videos of children during hospital-based interviews. They then analyzed these videos using OpenPose and applied ML to the data obtained. In this study, an accuracy of 91.03%, sensitivity of 90.25%, specificity of 91.86%, and an AUC of 94.00% were reported (34). In our study, the classification model distinguished between the ADHD and control groups with an accuracy of 81.82% and an AUC of 0.85. However, these studies differ substantially from the present work in terms of sensing modality, interaction context, and feature extraction strategies. Accordingly, direct performance comparisons should be interpreted cautiously.

Previous work examining movement patterns in ADHD has reported increased head movement relative to typically developing peers. For example, Demirdöğen et al. (11) found that children with ADHD showed greater head movements during a 5-minute classroom-style video task. In that design, head movement was the main measurable motor parameter, and increased head motion was interpreted in relation to attentional control. They found that children with ADHD also showed more saccadic shifts toward irrelevant areas. Although their sample included different ADHD presentations, no subtype-specific analyses were conducted. In our study, which included only the combined presentation and used a comparable short instructional paradigm, we similarly observed increased head movement in the ADHD group. Taken together, these findings point to a similar pattern of increased head movement in ADHD, though variations in methodology and sample composition warrant cautious interpretation.

Similarly, Ouyang and colleagues reported increased movement frequency in the thigh and shoulder regions in children with ADHD using video-based analysis in a clinical setting (34). In a classroom-based study using a Kinect depth-sensor system, Sempere-Tortosa et al. demonstrated greater whole-body movement in children with ADHD, with particularly pronounced effects for head movement (38). Although these studies differ in recording modality and task structure, they collectively suggest that elevated motor activity across multiple body regions is a recurring group-level characteristic in ADHD. In our study, we found increased movement in the head and across upper and lower limb segments in children with ADHD compared to typically developing controls during the semi-structured interaction paradigm (**Table 2**).

In previous work, Wehrmann and Müller (2015) reported no substantial correlations between video-derived activity scores and parental or clinician-rated hyperactivity, despite strong correspondence with observed physical movement (12). In contrast, a classroom-based motion capture study has reported that children with ADHD exhibit increased objectively measured movement alongside higher hyperactivity scores on clinical rating scales (38). Although that study did not report direct correlation coefficients, its results suggest a concordance between objective motion metrics and clinical ratings of hyperactivity. In our study, we observe a modest association between parent-reported hyperactivity and objectively quantified movement (**Figure 4**).We similarly observed a comparable magnitude of association when movement measures were normalized for height. However, given the small-to-moderate magnitude of this correlation, the findings should be interpreted with appropriate caution.

Beyond movement-based digital markers, we believe that future research should integrate multimodal physiological signals to strengthen both diagnostic precision and ecological validity. Recent work shows that electrodermal activity, heart rate variability, and skin temperature collected via wearable devices can help distinguish individuals with ADHD (39). These findings suggest that autonomic nervous system markers may provide information that complements movement-based features. At the same time, advances in biocompatible wearable materials now allow continuous, non-invasive monitoring of physiological and biochemical signals from biofluids (40). We therefore see strong potential in combining movement analytics with multimodal physiological sensing within an integrated wearable framework to move toward more comprehensive digital phenotyping approaches in neurodevelopmental conditions.

Body size should be considered when interpreting cumulative displacement measures. Larger body size and longer limb length may theoretically influence absolute movement estimates. In a previous study, video-based activity measures were associated with age and body size, indicating that developmental factors may influence movement estimates (12). In developmental samples, age is strongly associated with height, and hyperactivity symptoms have been shown to decrease with increasing age (41, 42). In the present study, age and height were positively correlated, while height showed modest inverse associations with the global activity index, consistent with developmental reductions in motor hyperactivity. To determine whether group differences could be explained by anthropometric variability, we conducted additional analyses controlling for height. Importantly, group membership remained a significant predictor of global activity after adjustment, and the overall pattern of regional differences was preserved. These findings suggest that the observed movement differences in our sample are unlikely to be driven solely by body size or physical growth.

POV cameras are well suited for capturing face-to-face interactions, while placing minimal burden on clinicians and participants. Compared with wearable sensors and fixed-camera systems, POV cameras offer methodological advantages, alongside trade-offs that require careful consideration. Wearable devices such as accelerometers provide high temporal resolution and enable precise quantification of movement in real-world settings (43). However, because they are physically attached to the body, participants’ awareness of the device may influence natural movement patterns (44). Fixed-camera systems allow full-body capture without participant burden (34). However, they typically require a predefined camera placement and room geometry, which can limit naturalistic interaction. In contrast, POV cameras enable dynamic capture of face-to-face interactions from the examiner’s perspective while minimizing environmental setup requirements (45). This configuration may better preserve ecological aspects of interpersonal engagement, although it introduces dependence on examiner positioning and movement stability. Importantly, the semi-structured interaction context likely influenced both the extracted movement features and the resulting classification patterns. During face-to-face engagement with an adult examiner, children may attempt to regulate overt or large-scale body movements, particularly those that are socially noticeable. In such contexts, motor restlessness may instead manifest through more subtle distal movements, such as adjustments of the hands, wrists, or feet. This context-dependent behavioral modulation may partly explain why distal limb features contributed more strongly to classification performance in our model. Accordingly, we interpret the identified discriminative features as context-dependent expressions of motor regulation rather than as generalized indicators of hyperactivity across all environments.

POV glasses have been used in a variety of studies in the literature. Metcalfe and colleagues demonstrated that POV recordings obtained in simulated clinical settings can be used in medical education (46). Edmunds and colleagues reported that POV recordings can be used to assess eye contact in children (16). Following this work, Chong and colleagues applied POV recordings to the measurement of eye contact, while Kayış and colleagues used POV glasses in diagnostic processes in children with autism and adults with depression (17, 45, 47). The present study extends this line of research by applying POV-based analysis to the objective assessment of ADHD in children aged 7–11 years.

### 4.3 Limitations

We acknowledge several limitations. The modest sample size, single-site recruitment, and absence of external validation may limit the generalizability of the machine learning findings. In addition, although feature importance and selection procedures were conducted within a nested cross-validation framework, the final 5-fold cross-validation was performed using the pre-selected feature set on the full sample. This approach may introduce feature pre-selection bias and potentially lead to optimistic performance estimates. Independent validation in larger, multi-site cohorts and fully nested model selection procedures will be essential before considering clinical applicability.

We did not directly examine the incremental value of the proposed movement-based metrics relative to established clinical scales. The proposed approach is intended to add an objective and observer-independent behavioral dimension to existing assessments. Although video-based measures capture real-time behavior during standardized interactions, their added contribution beyond traditional symptom ratings remains to be systematically evaluated in future studies.

We also used a controlled, semi-structured interaction paradigm. Although this design strengthened experimental control and standardization, it may not fully capture the complexity and variability of behavior observed in more naturalistic classroom or clinical settings. At the same time the protocol was conducted by a single trained researcher, which ensured procedural consistency but may have introduced subtle influences related to individual interaction style. Future studies including multiple examiners and more naturalistic contexts are needed to evaluate whether movement-based measures yield similar patterns across different interaction settings.

While anthropometric normalization may further refine movement-based features, we chose to focus on raw displacement measures. This allowed us to maintain methodological simplicity and enhance potential clinical scalability without requiring additional measurements. Similarly, although the pelvic-root reference frame improves stability, it may have attenuated large-scale trunk sway or global postural instability signals. Future studies incorporating limb-length–adjusted, as well as complementary coordinate systems, may help further clarify developmental influences on motor activity and better capture whole-body postural dynamics.

Moreover, several factors beyond ADHD may influence motor activity during interpersonal interactions, including anxiety, arousal level, sleep-related factors, temperament, and broader neurodevelopmental traits. These influences are not specific to ADHD and may reflect more general behavioral regulation processes. Although severe psychiatric comorbidities and psychotropic medication use were excluded and sessions were conducted during daytime hours, such factors were not systematically assessed in the present study. Accordingly, motor activity should therefore be interpreted as a dimensional behavioral marker rather than a specific indicator of ADHD pathology.

Although pose estimation provides an objective and scalable method for quantifying movement, it is not free from technical constraints. Because the camera was mounted on the examiner, the visual reference frame was inherently dynamic rather than fixed. Even subtle head movements by the examiner could lead to small shifts in viewpoint. As a result, parts of the child’s body—particularly distal limbs—may occasionally approach the edge of the frame or become briefly occluded. Such effects may introduce additional variability into displacement-based features, despite our use of landmark confidence thresholds to monitor tracking quality. Future studies may benefit from additional stabilization procedures or hybrid recording configurations to further reduce viewpoint-related measurement error.

Finally, the current sample included only children with the combined presentation of ADHD, which limits the generalizability of our findings across subtypes. Movement-based measures primarily capture hyperactivity-related behaviors and may be less sensitive to predominantly inattentive presentations. Emerging evidence from motion-tracking paradigms suggests that motor and executive control patterns may differ across ADHD subtypes (48). This highlights the importance of future studies directly comparing movement profiles across inattentive, hyperactive–impulsive, and combined presentations.

### 4.4 Conclusions

In this study, we demonstrated that video-based movement analysis obtained during a brief, semi-structured interaction can capture meaningful differences in motor activity between children with and without ADHD. By combining a standardized recording protocol with a semi-structured interaction paradigm, we were able to identify specific movement patterns and show the feasibility of group-level discrimination. Importantly, this approach does not require wearable sensors or task-heavy experimental setups, which may increase its feasibility in clinical and educational settings. Rather than serving as a diagnostic tool, the present findings support the potential of video-based movement analysis as an adjunctive, objective behavioral measure that may complement existing clinical assessment practices. Further validation in larger and more diverse samples is needed before broader clinical application can be considered.

## Data Availability

The raw data supporting the conclusions of this article will be made available by the authors, without undue reservation.

## Supplementary material

Supplementary Figure 1 Mapping of the 33 MediaPipe pose landmarks to the 15 composite anatomical points used in the analyses. The head centroid was computed from landmarks 0–10; hand centroids from distal hand landmarks (right: 18, 20, 22; left: 17, 19, 21); and foot centroids from heel and foot-index landmarks (right: 30, 32; left: 29, 31). All remaining landmarks (shoulders [11, 12], elbows [13, 14], wrists [15, 16], knees [25, 26], and ankles [27, 28]) were analyzed individually without aggregation. Hip landmarks (23, 24) were not analyzed as independent regions due to the pelvic root–based coordinate system.

Supplementary Table 1 Normality test results for movement variables.

Supplementary Table 2 Sensitivity analysis using height-normalized movement features.

Supplementary Table 3 Group differences in regional movement magnitude (Median [Q1, Q3] and Mann–Whitney U tests) using a 10-frame temporal averaging window.

Supplementary Table 4 Group differences in regional movement magnitude (Median [Q1, Q3] and Mann–Whitney U tests) using a 15-frame temporal averaging window.

Supplementary Table 5 Multiple linear regression model predicting global activity from group status (ADHD) and height.
