# Supplementary material for: Attention deficit hyperactivity disorder assessment through objective measures: POV glasses and machine learning approach
Source: Front Psychiatry. 2026 Mar 17;17:1785988. doi: 10.3389/fpsyt.2026.1785988 (PMC13035793; doi:10.3389/fpsyt.2026.1785988)

Figure S1. Mapping of the 33 MediaPipe pose landmarks to the 15 composite anatomical points used in the analyses. The head centroid was computed from landmarks 0–10; hand centroids from distal hand landmarks (right: 18, 20, 22; left: 17, 19, 21); and foot centroids from heel and foot-index landmarks (right: 30, 32; left: 29, 31). All remaining landmarks (shoulders [11, 12], elbows [13, 14], wrists [15, 16], knees [25, 26], and ankles [27, 28]) were analyzed individually without aggregation. Hip landmarks (23, 24) were not analyzed as independent regions due to the pelvic root–based coordinate system.

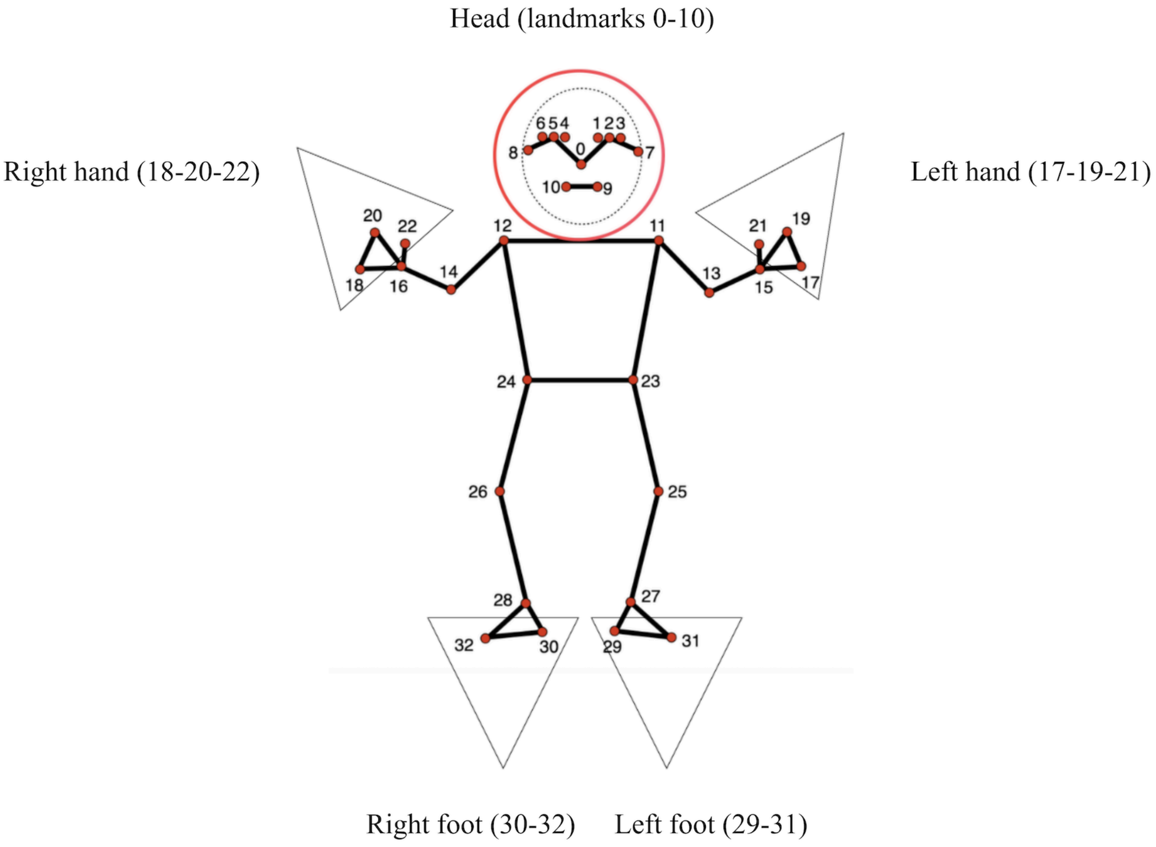

Supplement: Supplementary Figure 1 — Mapping of the 33 MediaPipe pose landmarks to the 15 composite anatomical points used in the analyses. The head centroid was computed from landmarks 0–10; hand centroids from distal hand landmarks (right: 18, 20, 22; left: 17, 19, 21); and foot centroids from heel and foot-index landmarks (right: 30, 32; left: 29, 31). All remaining landmarks (shoulders [11, 12], elbows [13, 14], wrists [15, 16], knees [25, 26], and ankles [27, 28]) were analyzed individually without aggregation. Hip landmarks (23, 24) were not analyzed as independent regions due to the pelvic root–based coordinate system. [file Image1.pdf]
