# Supplementary material for: Attention deficit hyperactivity disorder assessment through objective measures: POV glasses and machine learning approach
Source: Front Psychiatry. 2026 Mar 17;17:1785988. doi: 10.3389/fpsyt.2026.1785988 (PMC13035793; doi:10.3389/fpsyt.2026.1785988)
Supplement: Supplementary Table 1 — Normality test results for movement variables. [file Table1.docx]

**Table S1.** Normality test results for movement variables

| **Body Region** | **ADHD W** | **ADHD p** | **Control W** | **Control p** |
| --- | --- | --- | --- | --- |
| Global Activity | 0.961 | 0.212 | 0.957 | 0.272 |
| Head | 0.862 | <0.001 | 0.944 | 0.124 |
| Left shoulder | 0.916 | 0.008 | 0.851 | 0.001 |
| Right shoulder | 0.907 | 0.005 | 0.854 | 0.001 |
| Left elbow | 0.935 | 0.032 | 0.922 | 0.034 |
| Right elbow | 0.875 | 0.001 | 0.866 | 0.002 |
| Left wrist | 0.943 | 0.059 | 0.936 | 0.078 |
| Right wrist | 0.946 | 0.074 | 0.863 | 0.001 |
| Left hand | 0.939 | 0.044 | 0.930 | 0.056 |
| Right hand | 0.951 | 0.107 | 0.869 | 0.002 |
| Left knee | 0.945 | 0.068 | 0.923 | 0.036 |
| Right knee | 0.933 | 0.028 | 0.945 | 0.140 |
| Left ankle | 0.953 | 0.116 | 0.762 | <0.001 |
| Right ankle | 0.930 | 0.022 | 0.620 | <0.001 |
| Left foot | 0.921 | 0.012 | 0.717 | <0.001 |
| Right foot | 0.918 | 0.010 | 0.587 | <0.001 |
