# Supplementary material for: Attention deficit hyperactivity disorder assessment through objective measures: POV glasses and machine learning approach
Source: Front Psychiatry. 2026 Mar 17;17:1785988. doi: 10.3389/fpsyt.2026.1785988 (PMC13035793; doi:10.3389/fpsyt.2026.1785988)
Supplement: Supplementary Table 2 — Sensitivity analysis using height-normalized movement features. [file Table2.docx]

**Table S2.** Sensitivity analysis using height-normalized movement features

| **Body region** | **U** | **Z** | **p (2-tailed)** |
| --- | --- | --- | --- |
| Head | 284.0 | −3.26 | 0.001 |
| Left shoulder | 283.0 | −3.28 | 0.001** |
| Right shoulder | 281.0 | −3.30 | 0.001** |
| Left elbow | 288.0 | −3.21 | 0.001** |
| Right elbow | 284.0 | −3.26 | 0.001** |
| Left wrist | 390.0 | −1.89 | 0.058 |
| Right wrist | 373.0 | −2.11 | 0.035* |
| Left hand | 412.0 | −1.61 | 0.108 |
| Right hand | 393.0 | −1.85 | 0.064 |
| Left knee | 340.0 | −2.54 | 0.011** |
| Right knee | 340.0 | −2.54 | 0.011** |
| Left ankle | 305.0 | −2.99 | 0.003** |
| Right ankle | 307.0 | −2.97 | 0.003** |
| Left foot | 308.0 | −2.95 | 0.003** |
| Right foot | 295.0 | −3.12 | 0.002** |

**Note:** * p < 0.05 (uncorrected).

** Significant after domain-specific Bonferroni correction (upper limb α = 0.0125; lower limb α = 0.0167).

Head was tested without multiple-comparison correction.
