# Supplementary material for: Attention deficit hyperactivity disorder assessment through objective measures: POV glasses and machine learning approach
Source: Front Psychiatry. 2026 Mar 17;17:1785988. doi: 10.3389/fpsyt.2026.1785988 (PMC13035793; doi:10.3389/fpsyt.2026.1785988)
Supplement: Supplementary Table 3 — Group differences in regional movement magnitude (Median [Q1, Q3] and Mann–Whitney U tests) using a 10-frame temporal averaging window. [file Table3.docx]

**Table S3.** Group differences in regional movement magnitude (Median [Q1, Q3] and Mann–Whitney U tests) using a 10-frame temporal averaging window.

| **Body region** | **ADHD Median [Q1, Q3] (×10³)** | **Control Median [Q1, Q3] (×10³)** | **U** | **Z** | **Effect size (r)** | **p (2-tailed)** |
| --- | --- | --- | --- | --- | --- | --- |
| Head | 2.59 [1.93, 3.09] | 1.97 [1.56, 2.27] | 290 | −3.19 | 0.39 | 0.001 |
| Left shoulder | 1.99 [1.41, 2.57] | 1.49 [1.32, 1.69] | 295 | −3.12 | 0.38 | 0.002 ** |
| Right shoulder | 2.09 [1.46, 2.60] | 1.48 [1.32, 1.72] | 295 | −3.12 | 0.38 | 0.002 ** |
| Left elbow | 2.88 [2.19, 3.46] | 2.11 [1.80, 2.42] | 289 | −3.20 | 0.39 | 0.001 ** |
| Right elbow | 2.76 [2.08, 3.45] | 2.22 [1.64, 2.38] | 292 | −3.16 | 0.39 | 0.002 ** |
| Left wrist | 4.80 [3.78, 6.68] | 3.85 [2.78, 5.07] | 376 | −2.07 | 0.26 | 0.038 |
| Right wrist | 5.14 [3.44, 6.57] | 3.42 [2.70, 5.32] | 372 | −2.13 | 0.26 | 0.034 |
| Left hand | 5.58 [4.45, 7.84] | 4.64 [3.21, 6.07] | 403 | −1.73 | 0.21 | 0.085 |
| Right hand | 6.07 [4.03, 7.96] | 4.10 [3.31, 6.35] | 387 | −1.93 | 0.24 | 0.053 |
| Left knee | 3.81 [3.34, 4.77] | 2.97 [2.53, 3.94] | 339 | −2.55 | 0.31 | 0.011 ** |
| Right knee | 3.53 [2.97, 4.58] | 3.05 [2.33, 3.78] | 382 | −2.00 | 0.25 | 0.046 |
| Left ankle | 3.56 [2.52, 4.84] | 2.53 [1.95, 3.15] | 314 | −2.88 | 0.35 | 0.004 ** |
| Right ankle | 3.40 [2.86, 4.62] | 2.53 [1.94, 2.92] | 294 | −3.13 | 0.39 | 0.002 ** |
| Left foot | 4.56 [3.05, 5.76] | 2.81 [2.06, 3.59] | 304 | −3.00 | 0.37 | 0.003 ** |
| Right foot | 4.41 [3.28, 5.74] | 2.80 [2.19, 3.29] | 295 | −3.12 | 0.38 | 0.002 ** |

**Note:** * p < 0.05 (uncorrected).

** Significant after domain-specific Bonferroni correction (upper limb α = 0.0125; lower limb α = 0.0167).

Head was tested without multiple-comparison correction.

Effect size r was calculated as Z / √N (N = 66). Values around 0.1 indicate small, 0.3 medium, and ≥0.5 large effects.

Movement values are presented after multiplication by 10³ for readability; statistical analyses were conducted using the original values.
