# Supplementary material for: Attention deficit hyperactivity disorder assessment through objective measures: POV glasses and machine learning approach
Source: Front Psychiatry. 2026 Mar 17;17:1785988. doi: 10.3389/fpsyt.2026.1785988 (PMC13035793; doi:10.3389/fpsyt.2026.1785988)
Supplement: Supplementary Table 4 — Group differences in regional movement magnitude (Median [Q1, Q3] and Mann–Whitney U tests) using a 15-frame temporal averaging window. [file Table4.docx]

**Table S4.** Group differences in regional movement magnitude (Median [Q1, Q3] and Mann–Whitney U tests) using a 15-frame temporal averaging window.

| **Body region** | **ADHD Median [Q1, Q3] (×10³)** | **Control Median [Q1, Q3] (×10³)** | **U** | **Z** | **Effect size (r)** | **p (2-tailed)** |
| --- | --- | --- | --- | --- | --- | --- |
| Head | 3.10 [2.34, 3.75] | 2.46 [1.75, 2.80] | 284 | −3.26 | 0.40 | 0.001 |
| Left shoulder | 2.36 [1.59, 2.66] | 1.72 [1.45, 2.01] | 295 | −3.12 | 0.38 | 0.002 ** |
| Right shoulder | 2.34 [1.62, 2.74] | 1.70 [1.50, 2.05] | 299 | −3.07 | 0.38 | 0.002 ** |
| Left elbow | 3.37 [2.49, 4.24] | 2.49 [2.02, 2.85] | 290 | −3.19 | 0.39 | 0.001 ** |
| Right elbow | 2.99 [2.42, 4.33] | 2.60 [1.79, 2.83] | 303 | −3.02 | 0.37 | 0.003 ** |
| Left wrist | 6.09 [4.56, 7.89] | 4.61 [3.14, 6.02] | 362 | −2.25 | 0.28 | 0.024 |
| Right wrist | 6.06 [4.21, 7.48] | 4.16 [3.30, 6.02] | 370 | −2.15 | 0.26 | 0.031 |
| Left hand | 7.00 [5.28, 9.31] | 5.59 [3.69, 7.19] | 385 | −1.96 | 0.24 | 0.050 |
| Right hand | 7.09 [4.95, 9.22] | 4.90 [3.76, 7.89] | 386 | −1.94 | 0.24 | 0.052 |
| Left knee | 4.12 [3.50, 5.33] | 3.39 [2.79, 4.49] | 344 | −2.49 | 0.31 | 0.013 ** |
| Right knee | 3.96 [3.25, 4.98] | 3.31 [2.53, 4.42] | 378 | −2.05 | 0.25 | 0.041 |
| Left ankle | 4.13 [2.95, 5.20] | 2.75 [2.22, 3.65] | 315 | −2.86 | 0.35 | 0.004 ** |
| Right ankle | 3.91 [2.95, 5.33] | 2.80 [2.30, 3.41] | 317 | −2.84 | 0.35 | 0.005 ** |
| Left foot | 4.92 [3.39, 6.24] | 3.28 [2.19, 4.29] | 312 | −2.90 | 0.36 | 0.004 ** |
| Right foot | 4.83 [3.44, 6.47] | 3.24 [2.61, 4.10] | 307 | −2.97 | 0.37 | 0.003 ** |

**Note:** * p < 0.05 (uncorrected).

** Significant after domain-specific Bonferroni correction (upper limb α = 0.0125; lower limb α = 0.0167).

Head was tested without multiple-comparison correction.

Effect size r was calculated as Z / √N (N = 66). Values around 0.1 indicate small, 0.3 medium, and ≥0.5 large effects.

Movement values are presented after multiplication by 10³ for readability; statistical analyses were conducted using the original values.
