# Supplementary material for: Attention deficit hyperactivity disorder assessment through objective measures: POV glasses and machine learning approach
Source: Front Psychiatry. 2026 Mar 17;17:1785988. doi: 10.3389/fpsyt.2026.1785988 (PMC13035793; doi:10.3389/fpsyt.2026.1785988)
Supplement: Supplementary Table 5 — Multiple linear regression model predicting global activity from group status (ADHD) and height. [file Table5.docx]

**Table S5.** Multiple linear regression model predicting global activity from group status (ADHD) and height.

| **Predictor** | **B [95% CI]** | **SE** | **β** | **p** |
| --- | --- | --- | --- | --- |
| Intercept | 0.083 [0.034, 0.131] | 0.024 | — | 0.001 |
| Group (ADHD = 1) | 0.008 [0.002, 0.014] | 0.003 | 0.31 | 0.010 |
| Height (per 10 cm) | −0.003 [−0.010, −0.0002] | 0.002 | −0.24 | 0.046 |

**Note:** B = unstandardized regression coefficient; β = standardized coefficient. Height effect is reported per 10 cm increase. Model statistics: F(2, 63) = 6.97, p = .002, adjusted R² = .155.
